# Organoboron Compounds: Effective Antibacterial and Antiparasitic Agents

**DOI:** 10.3390/molecules26113309

**Published:** 2021-05-31

**Authors:** Paolo Saul Coghi, Yinghuai Zhu, Hongming Xie, Narayan S. Hosmane, Yingjun Zhang

**Affiliations:** 1 School of Pharmacy Macau, University of Science and Technology, Taipa Macau 999078, China; coghips@must.edu.mo; 2 State Key Laboratory of Quality Research in Chinese Medicine, Macau University of Science and Technology, Taipa Macau 999078, China; 3 The State Key Laboratory of Anti-Infective Drug Development (NO. 2015DQ780357), Sunshine Lake Pharma Co., Ltd., Dongguan 523871, China; xiehongming@hec.cn; 4 Department of Chemistry and Biochemistry, Northern Illinois University, DeKalb, IL 60115, USA

**Keywords:** organoboron compound, anti-cancer drug, anti-tuberculosis, anti-malaria drug, neglected tropical disease, crypto and toxoplasmosis treatment

## Abstract

The unique electron deficiency and coordination property of boron led to a wide range of applications in chemistry, energy research, materials science and the life sciences. The use of boron-containing compounds as pharmaceutical agents has a long history, and recent developments have produced encouraging strides. Boron agents have been used for both radiotherapy and chemotherapy. In radiotherapy, boron neutron capture therapy (BNCT) has been investigated to treat various types of tumors, such as glioblastoma multiforme (GBM) of brain, head and neck tumors, etc. Boron agents playing essential roles in such treatments and other well-established areas have been discussed elsewhere. Organoboron compounds used to treat various diseases besides tumor treatments through BNCT technology have also marked an important milestone. Following the clinical introduction of bortezomib as an anti-cancer agent, benzoxaborole drugs, tavaborole and crisaborole, have been approved for clinical use in the treatments of onychomycosis and atopic dermatitis. Some heterocyclic organoboron compounds represent potentially promising candidates for anti-infective drugs. This review highlights the clinical applications and perspectives of organoboron compounds with the natural boron atoms in disease treatments without neutron irradiation. The main topic focuses on the therapeutic applications of organoboron compounds in the diseases of tuberculosis and antifungal activity, malaria, neglected tropical diseases and cryptosporidiosis and toxoplasmosis.

## 1. Introduction

Many infectious diseases are caused by microorganisms, such as tuberculosis and malaria, and the current treatments for them are unsatisfactory as there are a few or no suitable drugs. Four types of such frequently occurring diseases, in which organoboron compounds have already shown high potential as acceptable drug agents, have been selected to survey in this review. The four common diseases are tuberculosis, malaria, neglected tropical diseases, and the parasitic diseases of cryptosporidiosis and toxoplasmosis, and they are briefly introduced as follows. (1) Tuberculosis is an infectious disease caused by *Mycobacterium tuberculosis*, which has a high level of mortality worldwide and has already gained resistance to first- and second-line therapy [1]. (2) Malaria is a disease caused by the *Plasmodium* parasite and accounts for one of the leading causes of death worldwide despite decades of strategic interventions aimed at reducing incidence and mortality [2]. (3) Neglected tropical diseases (NTDs) are a group of twenty highly parasitic (*fungi*, *protozoa*, *helminths* or metazoan worms), viral and bacterial diseases as classified by the World Health Organization (WHO) [2,3,4]. NTDs affect more than one billion people, especially children, and prevail in poor populations living in tropical and subtropical climates, causing a huge toll in terms of morbidity and mortality, as well as public economies [2,3,4], and (4) cryptosporidiosis and toxoplasmosis are other dangerous diseases caused by important protozoan pathogens of humans, while Cryptosporidium is a common cause of moderate-to-severe diarrhea in children under five years of age [5].

**Figure 1:**
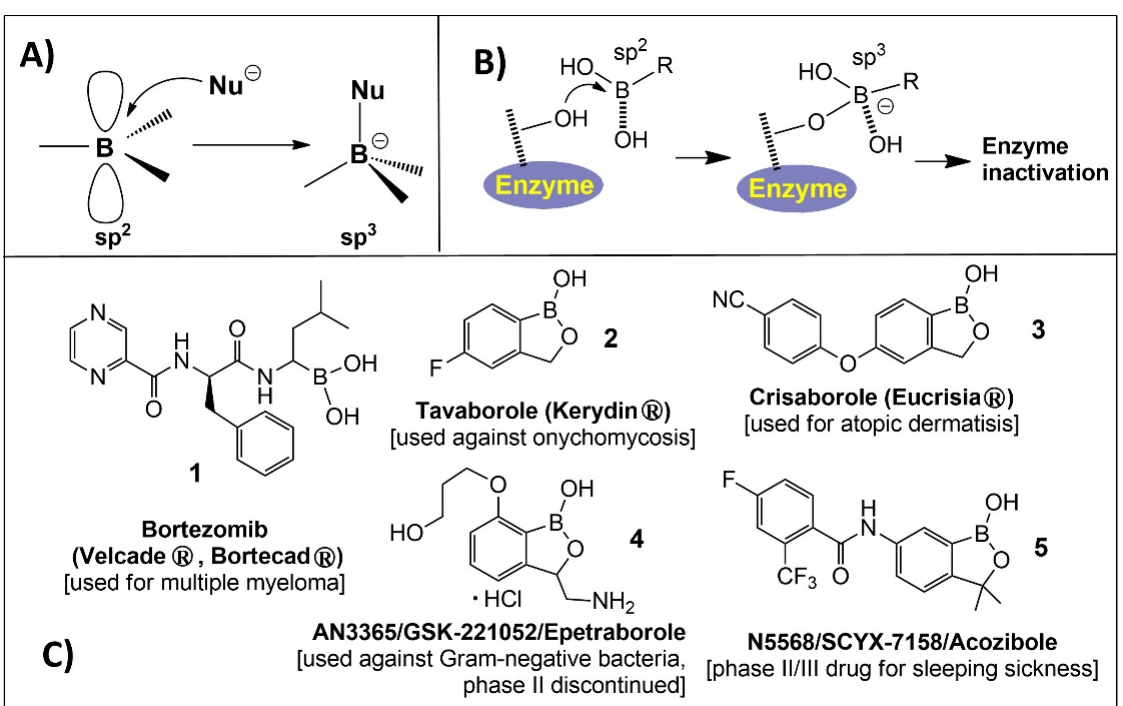
(**A**) Boron electronic features and configurational modification of boron; (**B**) Mechanism of action of boron-based compounds for enzyme inhibition; (**C**) Examples of reported boron compounds and marketed benzoxazole drugs.

Boron has a wide range of applications in chemistry, energy research, materials science and the life sciences [6,7,8,9,10,11]. The use of organoboron compounds as medication agents has a long history. For example, boron compounds, 4-borono-*L*-phenylalanine (BPA) and sodium borocaptate (BSH), have been used as boron carriers in boron neutron capture therapy (BNCT) for decades to treat various tumors, such as malignant brain tumor and melanoma [8]. Nevertheless, the updating of BNCT is beyond the scope of this review. In the beginning of the 20th century, many scientists concentrated their attention on the development of boron-based organic chemistry [9]. Cluster-based boron compounds are in the latest class that takes advantage of the properties of many boron atoms in the cage [10,11], including their unique electronic properties and ability to form covalent bonds in organoboron compounds, which make them a suitable agent for drug discovery. Boron compounds are electrophiles (strong Lewis acids) due to their empty *p*-orbital. When accepting a pair of electrons from a nucleophile (Lewis base), they easily inter-convert from the trigonal sp^2^ to the tetrahedral sp^3^ hybridization states, as shown in Figure 1A. The use of organoboron compounds as enzyme inhibitors is mostly based on this easy conversion (Figure 1B) [12]. After decades of studies, numerous bioactive molecules and molecular tools containing single boron atoms were developed [13]. Bortezomib, **1** (PS-341), (Figure 1C) trade name Velcade, from Takeda Pharmaceutical, is a dipeptide boronic acid (peptidomimetic), and it was approved by the FDA in 2003 for the clinical treatment of multiple myeloma [14]. Benzoxaboroles acquired reputation in medicinal chemistry only as of 2006, when 5-fluorobenzoxaborole (Tavaborole, AN2690, **2** in Figure 1C,) showed antifungal action [15], and its topical solution (Kerydin^®^) was approved by the FDA in 2014 for the treatment of onychomycosis [16]. Another benzoxaborole approved by the FDA in December 2016 was Crisaborole **3** (Figure 1C), which is traded in the USA under the name of EUCRISA^®^ for clinical use in the treatment of mild-to-moderate atopic dermatitis [17]. Currently, it is the first and only non-steroidal in anti-inflammatory monotherapy as the phosphodiesterase type 4 inhibitor, commonly referred to as a *PDE4* inhibitor for the skin. Benzoxaboroles possess unique chemical properties, such as remarkable chemical stability, low toxicity, ease in synthesis, and high targeting specificity. These attributes make them very attractive therapeutic agents [18]. In addition, several organoboron compounds also demonstrate strong antibacterial activity, specifically against the enteric group of Gram-negative bacteria. In this context, a promising example of an antibacterial oxaborole-based species is the chiral benzoxaborole **4** (AN3365/GSK2251052) (Figure 1C) [19]. Compound SCYX-7158/AN5568 (**5**, in Figure 1C) is identified as a promising agent for Human African trypanosomiasis (HAT) and has entered clinical phase II/III evaluation. Earlier observations of anti-fungal, anti-bacterial, and anti-inflammatory activities of benzoxaboroles and other organoboron compounds represented the key result that led to the discovery of their potential for the treatment of various infectious diseases [20]. This review will focus on the particular type of bioactivity of organoboron compounds covering the medicinal applications in infectious disease caused by *protozoa*, *fungi* and *helminths*, describing progress in drug development, cytotoxicity and the proposed mechanisms of action. Other organoboron compound-based antibacterial or antiviral drugs have been reviewed elsewhere [21,22]. Thus, the review covers four areas of therapeutic applications of organoboron compounds: tuberculosis and antifungal activity, malaria, neglected tropical diseases and cryptosporidiosis and toxoplasmosis.

## 2. Tuberculosis and Antifungal Activity

Tuberculosis (TB), caused by *Mycobacterium tuberculosis* (*Mtb*), is a highly contagious chronic bacterial infection and is one of the top 10 causes of death worldwide [23]. In 2019, more than 10 million people fell ill with TB, and around 1.4 million died from the disease [23]. The *Mtb* is transmitted by aerosol and infection occurs when a person inhales droplet nuclei containing tubercle bacilli that reach the alveoli of the lungs. These tubercle bacilli are ingested by alveolar macrophages and destroyed or inhibited. If the bacilli remain alive, they may spread by way of lymphatic channels or the bloodstream to other tissues and organs (brain, larynx, lymph node, lung, spine, bone, or kidney). Within 2 to 8 weeks, special immune cells called macrophages ingest and surround the tubercle bacilli. The cells form a barrier shell (granuloma) that keeps the bacilli contained and under control. If the immune system cannot keep the tubercle bacilli under control, the bacilli begin to multiply rapidly (TB disease) [24]. Worldwide, in 2019, close to half a million people developed rifampicin-resistant TB (RR-TB), of which 78% had multidrug-resistant TB (MDR-TB) [23]. MDR-TB is treatable and curable by using second-line drugs. However, second-line treatment (kanamycin, amikacyn) options are limited, and they require extensive chemotherapy (up to 2 years of treatment) with medicines that are expensive and toxic [25]. In this regard, many efforts have been dedicated to the discovery and development of new anti-TB agents with new mechanisms of action to control drug-resistant disease [26]. The most active frontiers are surviving as follows.

### 2.1. Benzoxaboroles

1,3-Dihydro-1-hydroxyl-2,1-benzoxaboroles (or dihydrobenzoxaborole or benzoboroxoles) were first synthesized and characterized in 1957 by Torssell [27]. After the discovery that *ortho-*hydroxyalkyl arylboronic acids can form a complex with glycosides under physiologically relevant conditions, they have been investigated as molecular receptors for sugars and glycoconjugates, in supramolecular chemistry and as building blocks and protecting groups in organic synthesis [28]. Reviews describing these applications of benzoxaboroles were recently published [29,30]. 

**Figure 2:**
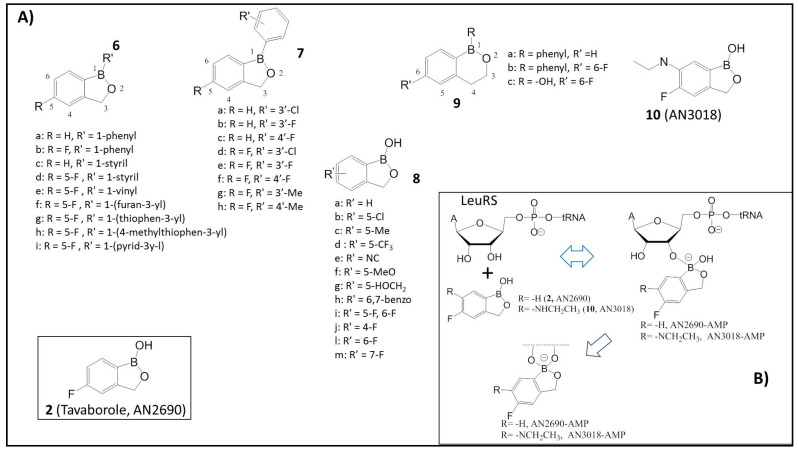
(**A**) Schematic representations of benzoxaborole compounds **2** (AN2690) and **6**–**10**; (**B**) Proposed reaction mechanism of **2** and **10** (AN3018) on leucyl tRNA synthetase (LeuRS) resulting in spiro-product inhibitor: The sp^2^ hybridized boron atom possesses an empty p-orbital that accepts electrons from the hydroxyl groups of the terminal adenosine and forms an adduct with the tRNA (Adapted from [31,32]).

The dihydrobenzoxaboroles bearing aryl, heteroaryl, or vinyl substituents at the 1-position (**6a–i**), as shown in Figure 2, were reported [29,30,31,32,33]. These substitutions showed equal or decreased activity against fungi. The first lead compound was 1-phenyldihydrobenzoxaborole, **6a**, which showed weak activity on a broad spectrum of fungi with minimum inhibitory concentration (MIC) values of 4–8 μg/mL. The following substitution to 5-fluoro-substituted benzoxaborole **6b** led to a 2- to 8-fold increase in antifungal activity. Starting from compound **6a** to determine the effect of hydrophobicity, many derivatives with various substitutions of R′ in the phenyl ring in position 1 (1-phenyldihydrobenzoxaborole **7a-h**) (Figure 2A) were synthesized. 

To enhance hydrophilicity, the 1-phenyl group was replaced with a 1-hydroxy group to prepare 1-hydroxydihydrobenzoxaboroles (**8a**), as per the published report. Compound **8a** showed an 8-fold increase in activity against *C. neoformans*, and **2** (AN2690) showed an 8-fold increase in activity against *A. fumigatus*, respectively [29,30,31,32,33]. To determine the structure–activity relationship of this scaffold, the 5-F group was substituted with other groups (**8b–m**). The results showed that **2** (R′ −F) and **8b** (AN2718, R′ −Cl) are the most active derivatives. The 5-chloro-substituted benzoxaborole **8b** (AN2718) is being developed now by Anacor pharmaceutical, a company pioneering the field of boron compounds, for the topical treatment of tinea pedis, dermatophyte fungal infection of the soles of the feet and the interdigital spaces [29,30,31,32,33]. The ring size increase from a five-membered oxaborole of **6a**, **6b**, and **2** to the corresponding six-membered oxaborin **9a**, **9b** and **9c** showed that 1-phenyl substituted oxaborin **9a** and the 5-fluoro-1-phenyloxaborin **9b** were approximately 2-fold and 4- to 16-fold less active than the oxaborole **6a** and **6b**, respectively [29,30,31,32,33].

Compound **2** is the most active against dermatophytes *T. rubrum* and *T. mentagrophytes*, which are the primary fungal pathogens that cause onychomycosis [31]. The FDA approved the application of **2** (Tavaborole, AN2690) in 2014 as the first oxaborole-based antifungal new drug for the topical treatment of onychomycosis of the toenails [34]. The mechanism involves Tavaborole **2** forming a covalent adduct with the 3′-adenosine of tRNA and inhibiting leucyl-tRNA synthetase (LeuRS) (IC_50_ 2.1 μM) (Figure 2B) [32]. LeuRS belongs to aminoacyl-tRNA synthetases (aaRS), a class of enzymes which are crucial for gene translation. It also plays a critical role in protein synthesis by catalyzing the specific amino acid attachments to their corresponding tRNA during the translation of the genetic code. Many aaRS enzymes possess a proofreading (editing) mechanism that hydrolyzes tRNAs’ aminoacylated functionality with the incorrect amino acid [32]. Thus, LeuRS is a proofreading aaRS, which possesses distinct synthetic aminoacylation and editing active sites separated by more than 30Å. The aminoacylation reaction occurs in two steps: the formation of an enzyme-bound aminoacyl-adenylate (I), followed by the transfer of this activated amino acid to either the 2′- or 3′-hydroxy group on the 3′-terminal adenosine of tRNA (II) [32]. The inhibition of either one of these enzymatic stages (I, II) leads to the accumulation of uncharged tRNA molecules, which bind to ribosomes, causing the interruption of polypeptide chain elongation [32]. These enzymes have been a focus of antimicrobial research as potential targets for more than a decade [35]. Seiradake et al. determined the structure of the *C. albicans* editing domain complex with compound **10** (AN3018, 6-ethylamino analogue of **2**, Figure 2) to provide a structural basis for designing and enhancing the specificity of these benzoxaborole antifungals [36]. 

**Figure 3:**
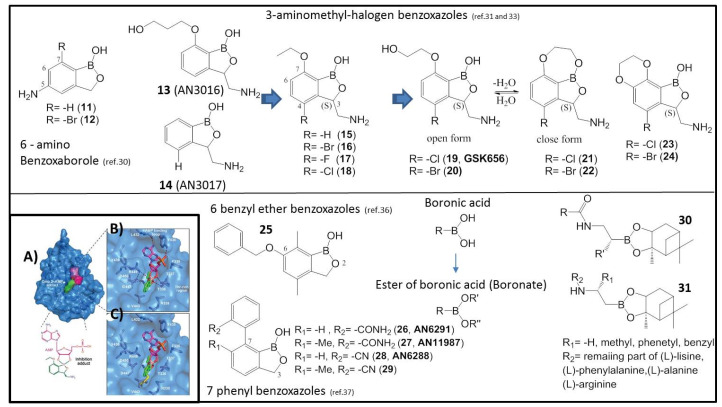
Structures of benzoxaborole compounds **11**–**31**. (**A**) X-ray cocrystal structure of LeuRS with compound **15.** Crystal structure of the *Mtb* LeuRS editing domain in complex with compound **15** (carbon atoms are shown in green) and AMP (carbon atoms are shown in magenta); (**B**) Zoomed view into the editing site of *M. tuberculosis* LeuRS showing the compound **15**-AMP adduct; (**C**) Overlay of the LeuRS editing domain of *Mtb* and *E. coli* in complex with methionine (in yellow). The 3-aminomethyl group of compound **15** mimics the amino group of methionine, including the interaction with the bacterium-specific residue D447. (Adapted from [36,37,38,40,45]).

The 6-aminobenzoboroxoles have also been synthesized and found to be non-toxic [37] in general. The derivatives of **11** and **12** with primary amino groups showed good antimycobacterial activity against *Mtb* H37Rv (**11**, MIC 1.9 μM, **12** MIC 15.6 μM) [37]. The study identified two lead compounds of **11** and **12**, which urges their further development. In the course of an initial drug screening, conducted in Anacor Pharmaceuticals, benzoxaboroles **13** (AN3016) and **14** (AN3017) were found to provide low MIC against *Mtb* H37Rv (**13**, 1 μg/mL and **14**, 1.8 μg/mL) and inhibitory activity towards *Mtb* LeuRS (IC_50_: **13**, 3.5 μM; **14**, 0.64 μM) [38]. The incorporation of 3-aminomethyl and 7-ethoxy moieties into one molecular structure to form compound **15** showed an increase in activity (**15**, MIC 0.13 μg/mL, *Mtb* LeuRS IC_50_ 0.13 μM) [38]. To improve the bio-capacity of **15**, structural and biophysical studies were performed through pharmacokinetic investigation to understand its binding mode to *Mtb* LeuRS. Crystallization with different editing domain constructs of *Mtb* LeuRS was attempted in the presence of compound **15** with AMP. The boron atom in compound **15** forms a bidentate covalent adduct with AMP (Figure 3A), which mimics the terminal nucleotide Ade76 of the tRNA acceptor. The amino acid residues of T336 to T337 of the threonine-rich region provide multiple H-bonding interactions to the covalent adduct, and L432 and Y435 of the AMP binding loop have extensive H-bonding and hydrophobic contacts with AMP (Figure 3B) [38]. In addition, the amino group of compound **15** builds three key interactions with the carboxylic acid side chains of D447 and D450 and the carbonyl group of M441. The 7-ethoxy substitution not only enables a new interaction with R449 but also packs with the Ade76 ribose, thus further stabilizing the boron-tRNA adduct (Figure 3B) [38]. The superposition of the adduct-bound structure of **15** with that of the *E. coli* LeuRS editing with the methionine-bound domain shows that the 3-aminomethyl benzoxaborole moiety occupies the same position as the non-cognate amino acid (Figure 3C) [39]. A series of 3-aminomethy benzoxaboroles were evaluated as *Mtb* LeuRS inhibitors, and most of them were produced and tested as a race mate first, and later separated into the active *(S)*-isomer. In general, the *(S)*-enantiomer is more potent compared to the race mate or A *(R)*-isomer [38]. Thus, the most potent analogs were compounds **16**–**18** with halogen (Br, F, Cl, (Figure 3)) substitutions at 4-position. These compounds showed an increase in activity against *Mtb* H37RV (MIC 0.02–0.05 μM), an increase in potency towards *Mtb* LeuRS (IC_50_ 0.06–0.08 μM) and, therefore, they were selected for in vivo murine pharmacokinetic analysis. All three compounds were very efficacious, in both the acute and chronic mouse *Mtb* models, with a potency comparable to that of the frontline drug isoniazid [38]. 

One of the major drawbacks of the series was its potential toxicity. In order to improve the selectivity of the *Mtb* LeuRS inhibitors, further studies were performed. First, lipophilicity optimization of the sidechain was investigated by incorporating aromatic moieties to the 7-alkoxyl group, but these derivatives showed a reduction or loss of antitubercular activity and a decrease in *Mtb* LeuRS potency. The introduction of one or two fluorine in the sidechain resulted in a slight decrease or similar antitubercular activity [40]. Subsequently, by increasing the hydrophilicity of the sidechain and reducing the linker to two-carbon in 7-position, the increase in activity of compounds **19** and **20** against *Mtb* LeuRS (**19**-GSK656, IC_50_, 0.20 μM; **20**, 0.12 μM) [40] was achieved. Existing equilibrium between an open and a closed form of **19**–**21** and **20**–**22** of the benzoxaborole pharmacophore has a dependency on solvent and environment [41]. In addition, the ring-fused compounds of **23** and **24** exhibited enhanced anti-tubercular activity against *Mtb* H37Rv with the MIC of 0.08 μM and 0.03 μM, respectively, and potent *Mtb* LeuRS activity of IC_50_ of 0.046 μM and 0.12 μM for **23** and **24**, respectively [41]. Compounds **19** and **23** exhibited low clearance and excellent exposure in drug metabolism and pharmacokinetics studies. The typical *Mtb* LeuRS inhibitor shows low molecular weight, low polar surface area (PSA), and clogD7.4 value similar to frontline *Mtb* drugs of isoniazid, pyrazinamide, and ethambutol [40]. 

To evaluate the ability of these *Mtb* LeuRS inhibitors for tuberculosis, treatment tests were conducted in vivo using an animal model. Compound **19** showed the best efficacy with an ED_99_ (efficacious dose that gives a two log colony-forming units (CFU) reduction compared to the untreated control) of 0.4 mg/kg among the evaluated compounds. For the best profile, with excellent in vivo efficacy at low doses in acute and chronic mouse TB infection models, compound **19** has been progressed to clinical development for the treatment of tuberculosis, the first time in Human (FTIH) safety and pharmacokinetics (PK) study of GSK3036656 in Healthy Subjects [42].

Patel et al. identified a novel 6-benzyl ether benzoxaborole **25** with potent activity against *Mtb* in vitro (MIC 2 μM), which was active against intracellular bacteria (50% inhibitory concentration IC_50_ 3.6 μM) with no cytotoxicity; thus, the profile of this compound is also encouraging for future development [43]. Meanwhile, a series of novel 7-phenyl benzoxaboroles were also investigated, where compounds **26**–**29** showed reasonable activity against *Mtb* in vitro (5.1–80 μM) with lower MIC_99_ (the concentration required to inhibit growth by 99%) (5–12.5 μM) [44]. These compounds may target NADH dehydrogenase (Ndh) rather than LeuRS [44]. Ndh is an essential oxidoreductase, which catalyzes the electron transfer process from NADH to menoquinone as part of the electron transport chain [45], and mutations in Ndh, found in clinical isolates, have shown resistance to isoniazid [46]. Further studies revealed that these processes correspond to residues involved in the quinone binding pocket [47]. This series of compounds shows potential for further development and to target validation work. In addition, dimeric benzoboroxoles were reported recently, and they were found to possess excellent selectivity and activity for mycobacteria, including the *Mtb* pathogen, and were capable of complexing to *Mtb* glycans without resistance [48].

### 2.2. Peptidyl Boronates/Boronic Acids

Boronates may interact with a target protein through covalent bonding with nucleophilic entities (such as hydroxyl and amine groups of enzymes, Figure 1B) to form a stable bond with the enzymes, thereby leading to their reversible inhibition. The boronic acid species can be incorporated with a peptide to form the corresponding peptidyl boronate/boronic acid, which may exhibit various biological activities [49,50]. Bortezomib (Takeda Pharmaceutical) (**1**, Figure 1C), trade name Velcade, is a dipeptide boronic acid and is the first human proteasome (H. proteasoma) inhibitor approved by the U.S. FDA for the treatment of multiple myeloma [51]. The X-ray crystal structure of the proteasome in a complex with bortezomib displayed a covalent bond formation between the boronic acid moiety of **1** and the hydroxyl group of Thr1 at the chymotrypsin-like active site of the 20S proteasome, leading to enzyme dysfunction and apoptosis in cancer cells [52,53] (H. proteasome IC_50_ 0.005 µM). However, bortezomib presented major drawbacks, such as high costs and poor pharmacokinetics with significant side effects (peripheral neuropathy, neutropenia, and cytopenia) despite its use to treat many cancers successfully [54].

**Figure 4:**
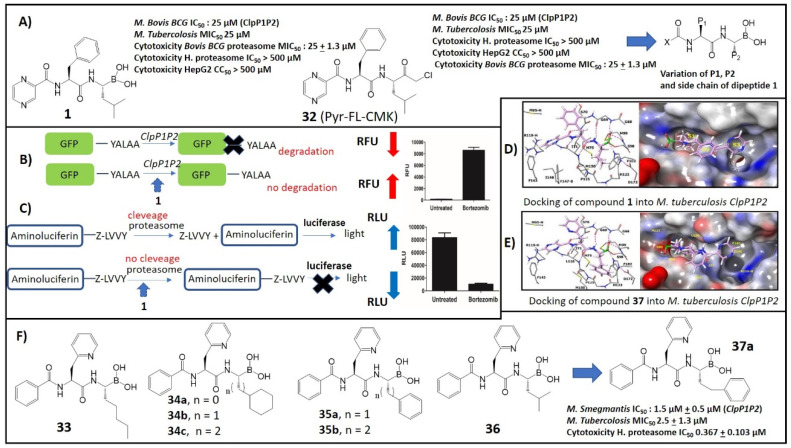
(**A**) Structures and antifungal activity of selective Mycobacterial *ClpP1P2* inhibitors **1** (Bortezomib) and **32**. (A series of dipeptidyl boronates with variation at the P1, P2, and X side-chains were synthesized); (**B**) *ClpP1P2* inhibition assay; (**C**) Proteasome inhibition assay; (**D**) Docking of **1** into *Mtb ClpP1P2*; (**E**) Docking of **37a** into *Mtb ClpP1P2*; (**F**) Structures and antifungal activity of selective Mycobacterial *ClpP1P2* inhibitors **33**–**37a** (Adapted from [59,62]).

Caseinolytic proteases (*ClpP*) are serine proteases found in a wide range of bacteria, and they have the ability to remove the aborted translation products [55]. The tmRNA trans-translation system, a bacterial rescue system that frees ribosomes stuck during protein synthesis, tags partially synthesized proteins with a caseinolytic-protease-specific (SsrA) degradation peptide. The SsrA-tagged proteins are recognized by the *ClpP* and degraded [56,57]. Mycobacteria, including *Mtb* and *Mycobacterium smegmatis*, encode two *ClpP* homologs, *clp*P1 and *clp*P2, in a single operon which associate together to form a single proteolytic complex, referred to as *ClpP1P2.* The caseinolytic protease complex is composed of catalytic protease subunits (*ClpP*) and regulatory subunits (ATPases). Both proteins are required for viability in vitro and during infection, and depletion of either protein results in the rapid death of the bacteria [58]. Genetic studies also suggest *ClpP* may serve as an ideal target for antimycobacterial therapy because of the synergistic nature of *ClpP1P2* protease depletion with mistranslation-inducing aminoglycosides that are important second-line drugs for *Mtb* [58]. Compound **1** was identified as a whole-cell-active *ClpP1P2* protease inhibitor in mycobacteria and a new lead compound for TB (*M. Bovis* IC_50_ *ClpP1P2,* 1.6 ± 0.5 µM, *M. Smegmatis* MIC_50_ 6 µM) through the mechanism-based whole-cell screening method from a library of over 500000 compounds (Figure 4A) [59]. To measure the intracellular *ClpP1P2* inhibition, Dick et al. engineered an *M. smegmatis* through screening that allows the detection of inhibitors of intracellular *ClpP1P2* activity via the accumulation of SsrA-tagged green fluorescent protein (GFP) (Figure 4B). In normal conditions, the *ClpP1P2* complex recognizes the SsrA- (YALAA) tagged with GFP (GFP-SsrA) and degrades the proteins, resulting in low basal fluorescence. In the presence of an inhibitor (Bortezomib, **1**), *ClpP1P2* binds to the catalytic sites of the protease and prevents the degradation of the GFP-SsrA proteins, resulting in its accumulation and a gain of fluorescence signal (Figure 4B) [59]. On the other hand, mammalian proteasome intracellular inhibition was measured using the whole-cell target-based proteasome-Glo assay, as shown in Figure 4C. This assay is based on a proteasome-specific cleavage tag (Z-LLVY) that fuses to an aminoluciferin molecule. In normal conditions, the proteasome cleaves the LLVY tag, allowing the luciferase to oxidize the aminoluciferin generating luminescence. In the presence of a proteasome inhibitor, the LLVY cleavage does not occur. The tagged aminoluciferin cannot be used by the luciferase enzyme, which is preventing the subsequent emission of luminescence [60]. 

Chloromethyl ketones (CMKs) comprise a distinct class of covalent irreversible serine protease inhibitors [61]. The function of this class of peptide is mechanistically similar to that of boronic acids. Compound **32** (pyrazine-phenylalanine-leucine-chloromethylketone), an analog of **1** containing a chloromethyl ketone (CMK) instead of the boronic acid, was synthesized and its potencies against the bacteria and human enzymes were determined [61]. Compound **32** retained its activity against mycobacterial *ClpP1P2* (IC_50_: 25 µM), against bactericidal *Mtb* (IC_50_: 25 µM) and was active against the mycobacterial proteasome (MIC_50_: 25 ± 1.3 µM), but was found to be devoid of activity against the mammalian human proteasome (IC_50_: >500 µM vs. **1** IC_50_: 0.005 µM) [61]. The CMK analog was not toxic to HepG2 cells at a concentration of up to 500 μM, while bortezomib displayed a cytotoxicity CC_50_ of 250 µM [61]. These chloromethyl compounds similarly inhibited both *ClpP1P2* and the proteasome in the bacteria while leaving the human proteasome untouched. These results suggest that the selectivity over the human proteasome is achievable [61]. Based on these results, a series of dipeptidyl boronate derivatives of **1**, with variation at the P1, P2, and X sidechains, were synthesized with a goal to identify compounds which inhibit bacterial *ClpP1P2* in a bacterial cell and have reduced potency against the human proteasome compared to bortezomib (Figure 4A) [62]. Replacing the *iso*-butyl group in P1 of **1** with a less hindered straight-chain *n*-pentyl (compound **33**, Figure 4F) increased the activity against *Mtb* twofold, whereas it decreased the potential in the proteasome assay by 6-fold (IC_50_: 0.03 µM) [62]. Aromatic derivatives of **35** showed 10–14-fold-lower potency for the proteasome compared to **1** [62]. Subsequent studies showed that a bulky group (benzyl and phenyl) in position X could increase the *ClpP1P2* inhibitory activity without a reduction in proteasome activity. Different bulky heterocyclic groups were also screened, and among them compound **36** with the 3-pyridyl group provided an interesting result of 6-fold-lower potency for the proteasome compared to **1** with retention of *ClpP1P2* inhibitory activity [62]. This series of changes of X offers options for subsequent P1–P2–X combinations for the future phase of SAR exploration. 

Docking studies suggested a larger P1 ligand could be accommodated in the P1 pocket of the *ClpP1P2* but less well tolerated in the P1 pocket of the human proteasome (Figure 4D). The docking of **37a** to the binding site of *ClpP1P2* indicates that the hydrophobic S1 residues Ile71, Met75, Met99, Phe102, and Pro125 interact with P1 (phenethyl group). Hydrogen bonds are also formed between the P2 amine and the backbone carbonyl of Leu126 and between the carbonyl of the N-terminal and the backbone amine of Ile71 (Figure 4E) [62]. In medicinal chemistry, the “drug likeness” of this selected compound was commonly investigated and predicted from its pharmacokinetic properties. Physicochemical properties such as molecular weight, numbers of hydrogen bond donors and acceptors and lipophilicity (LogP) were examined according to Lipinski’s rule of five [63]. Compound **37a** was selected for further profiling in vitro ADME assays (absorption, distribution, metabolism, and excretion). It had favorable in vitro ADME properties: plasma protein binding and human liver microsome stability was moderate, clearance in mouse microsomes was high (8min), and the inhibition of cytochrome P450 enzymes was not detected at the highest concentration tested. The Oral/i.v. pharmacokinetics of **37a** indicated moderate clearance and low bioavailability [62,64]. Therefore, ClpP1P2 inhibitors are a possible new strategy for the management of drug-resistant *M. Tubercolosis*.

### 2.3. Other Small Compounds of Boron (Diazoborines, Antibiotic)

**Figure 5:**
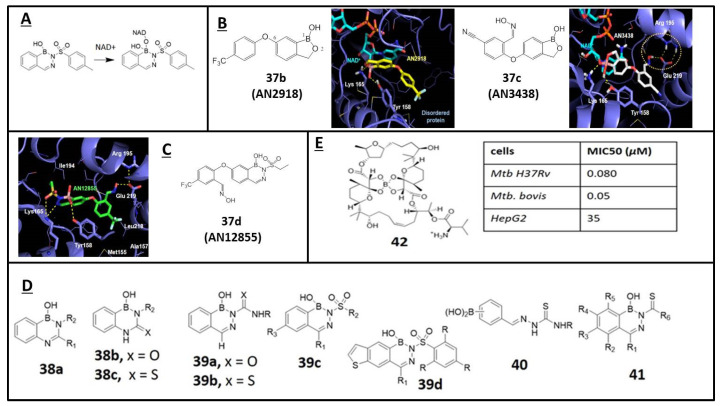
(**A**) Formation of a covalent B–O bond of diazaborines with the OH group at C (2′) of the NAD cofactors ribose unit of enzyme ENR; (**B**) Structure of oxaborole **37b** (AN2918) and **37c** (AN3418) and their complex crystal structure with *Mtb* InhA; (**C**) Structure of diazoborine **37d** (AN12855) and its complex crystal structure with *Mtb* InhA; (**D**) Structures and antifungal activity of 2,3,1-benzodiazaborines **38**–**41**; (**E**) Structures and cytotoxicity activities of Boromycin **42** (Adapted from [68,70,71,72,73]).

Diazaborines are a family of boron-containing compounds, in which the boron atom is stabilized in the form of an aromatic boron-based heterocycle. The antibacterial activities of 1,2-dihydro-l-hydroxy-2-(organosulfonyl)arenol-[*d*]-[1,2,3]-diazaborines are well documented in the literature [65]. It has been proposed that the mechanism of action of diazaborines in *E. coli* is by the complexation of nicotinamide adenine dinucleotide (NAD+) and the inhibition of enoyl-reductase (ENR) [66]. Similar to the benzoxaboroles such as **37b** (AN2918) and **37c** (AN3418), diazaborine inhibitors of ENR were found to form a covalent B–O bond with the OH group at C (2′) of the NAD cofactors ribose unit (Figure 5A,B) [67,68]. Mycobacteria have a similar enzyme with enoyl-reductase, InhA (Enoyl-[acyl-carrier-protein] reductase [NADH]), which is required for mycolic acid biosynthesis [69]. Recently, diazoborine **37d** (AN12855), which exhibited in vitro bactericidal activity against replicating bacteria, was revealed to inhibit the substrate-binding site of InhA in a novel cofactor-independent manner (IC_50_: InhA 0.03 µM, Figure 5C) [68]. 

Martin et al. first reported the synthesis of 2,4,1-benzodiazaborine compounds **38a–c** (R_1_= -pyrazinyl/R_2_ -H, -^n^Bu, -pyridyl), showing potent inhibitory activity against *M. tuberculosis* (Figure 5D) [70]. Subsequently, a set of 2-acylated 2,3,1-benzodiazaborines **39a–d** was synthesized, characterized, and tested with *Mycobacterium smegmatis* (Figure 5D) [71]. In addition, 2-formylphenyl boronic acids **40** (R= H, allyl, Ph) and their derivatives of **41** were also reported as potential antifungal agents, and their activity was examined against four fungi (*Aspergillus niger*, *Aspergillus flavus*, *Candida albicans*, and *Saccharomyces cerevisiae*) using Amphotericin B as a control and showed appreciable activity [72,73]. Boromycin **42** is a boron-containing polyether macrolide antibiotic isolated from *Streptomyces antibioticus*. It is a potent inhibitor of mycobacterial growth (MIC_50_: 80 nM) with strong bactericidal activity and low cytotoxicity vs. HepG2. It acts as an ionophore and causes the collapse of the potassium gradient across the bacillus’ membrane (Figure 5E) [74].

## 3. Malaria

Malaria, a parasitic infection by the Plasmodium genera, is spread through the bites of infected mosquitoes *Anophele*. It was responsible for an estimated 229 million clinical cases and 409,000 deaths worldwide in 2019, mostly among children under the age of five years [75]. Malaria is transmitted by parasites of the Plasmodium genus with five species known to infect humans: *P. falciparum*, *P. malariae*, *P. vivax*, *P. ovale* and *P. knowlesi*, with infections by *P. falciparum (Pf.)* and *P. vivax* being the most virulent [76]. Human malaria infection is initiated when a female anopheles mosquito deposits “sporozoites” during a blood meal. These sporozoites migrate to the liver where they undergo further development into schizonts, which produce “merozoites” that enter into the systemic circulation where they infect red blood cells and cause the typical symptoms of malaria. Some merozoites in these cells may develop into an asexual form called “trophozoites”, and in some cases into sexual forms of the parasite, called “gametocytes”, that circulate into the bloodstream. When a mosquito bites an infected human, it ingests the gametocytes, which develop further into mature sex cells called “gametes”. In the mosquito’s stomach, the male microgametes penetrate the female macrogametes, generating “zygotes”. The zygotes invade the midgut wall of the mosquito where they develop into “oocysts”. The oocysts grow, rupture, and release “sporozoites” which enter mosquito’s salivary glands. The inoculation of the sporozoites into a new human host will start a new malaria life cycle [77]. Chloroquine (CQ) was one of the most widely used antimalarial drugs, which has been now substituted by artemisinin (ART) and its synthetic derivatives [78]. The successful exploitation of semisynthetic ART derivatives was a major breakthrough in malaria chemotherapy because of their profound and rapid therapeutic response against malaria parasites. The WHO recommends that deadly species *P. falciparum* should be treated with artemisinin-based combination therapies (ACT), in which the ART-based component is combined with a second, longer-acting partner drug. However, reports of decreased efficacy, reduced parasite clearance time in the case of ACT treatment and widespread resistance by *Plasmodium* parasites [79,80] suggest the need for a new search for novel pharmaceutical interventions for malaria [81].

**Figure 6:**
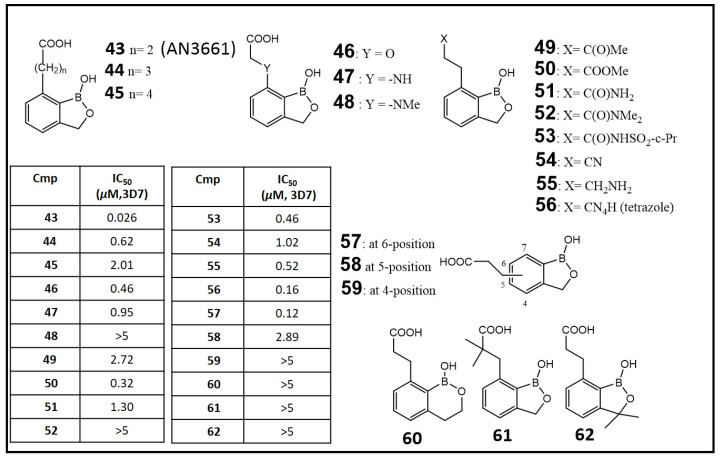
Structures and antimalarial activity (IC_50_) of benzoxaborales (**43**–**62**) against the malaria parasite *Plasmodium falciparum* CQ-sensitive 3D7 strain. (Adapted from [82]).

Early observation of antifungal, antibacterial and anti-inflammatory activities of benzoxaboroles led to the discovery of their potential for therapy of protozoan disease such as malaria, human African trypanosomiasis (HAT) and Chagas disease [81]. After screening in a whole cell assay against the malaria parasite *P. falciparum* of a boron-containing compound collection, Zhang et al. reported some potent hits (Figure 6), including 7-(2-carboxyethyl)-1,3-dihydro-1-hydroxy-2,1-benzoxaborole (**43**, AN3661, IC_50_: 0.026 μM). A series of analogs of 43 were designed to assess the structural features required for potent antimalarial activity, including the length of the sidechain on the oxaborole nucleus (**44**, **45**), the sidechain functional groups (**46–56**), the attaching positions of the sidechain (**57–59**), and modifications to the benzoxaborole scaffold (**60–62**) (Figure 6) [82,83]. Further structural modification, such as the introduction of fluoro, phosphonic and hydroxamic groups, was found to decrease the activity potency dramatically; it was also that the removal of the boron atom from the five-membered oxaborole ring reduced the antimalarial activity [84]. 

**Figure 7:**
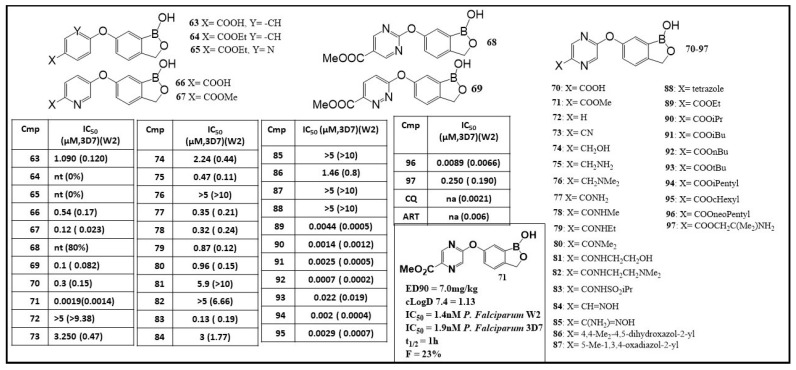
Structures and antimalarial activity (IC_50_) of benzoxaboroles (**63–97**) against the malaria parasite *Plasmodium falciparum* CQ-sensitive 3D7 and CQ-resistant W2 strains. (Adapted from [85]).

A new scaffold of 6-(4-carboxyphenoxy)-1,3-dihydro-1-hydroxy-2,1-benzoxaborole, **63** (shown in Figure 7), was identified in 2015 with a potent activity of IC_50_ of 0.120 μM against *P. falciparum* [85]. Further studies of structure–activity relationship (SARs) were performed by varying the 6-aryloxy group (**64**–**71**), substituent modification on the pyrazine ring (**72**–**88**) and exploring the effect of side-chain ester group (**89**–**97**) (Figure 7). To examine the effect of the left-side aromatic moiety on antimalarial activity, compounds **64**–**71** were designed. If the nitrogen was in an *ortho*-position to oxygen as in **65**, the incorporation of a nitrogen atom did not improve the activity, whereas nitrogen located at the meta-position to oxygen would increase the activity of **66** and **67** [85]. A pyrazine ring-embedded compound (**71**) showed extremely high potency both for *Pf* W2 strain and *Pf* 3D7 (IC_50_: 0.0014 μM for *Pf* W2; IC_50_: 0.0019 μM for *Pf* 3D7) [85]. With the identification of the pyrazine ring, compounds **72**–**88** were synthesized to investigate the effects of the substituent group in the ring. None of the compounds showed antimalarial activity better that **71**, and that would indicate the presence of the carboxylic ester as a crucial functionality. Other ester compounds (**89**–**97**) were designed and synthesized to further explore the effects of different esters (Figure 7). Compound **92** containing *n-*butyl ester showed outstanding potency with an IC_50_ value of 0.0002 μM for *Pf* W2 and 0.0007 μM for *Pf* 3D7, respectively [85,86]. Compound **71** demonstrated excellent efficacy in vivo against *P. berghei* in infected mice (ED_90_ 7.0 mg/kg). Nevertheless, the metabolic instability and less favored PK parameters of **71**, such as relatively short half-life and low bioavailability, warrant its further optimization (Figure 7, panel A) [85,86].

**Figure 8:**
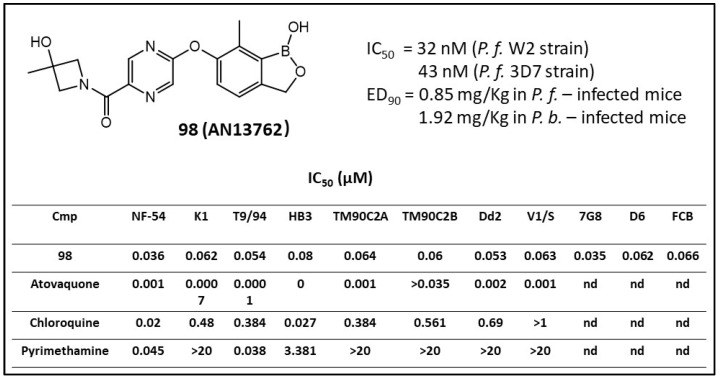
In vitro activities of **98** against multiple *P. falciparum* parasite strains (IC_50_) (Adapted from [86]).

To optimize the potency, stability and PK profile of such benzoxaborole derivatives, various carboxamide functional groups were incorporated. Analogues to different 1′-monoalkyl substituents in the amide sidechain had potencies ranging from 0.031 to 1.99 µM against *Pf* CQ-sensitive strain 3D7 and showed that the (R-) enantiomers were generally little more active than the (S-) isomers [86]. Among the screening compounds, compound 6-(2-((3-hydroxy-3-methylazetidin-1-yl) carbonyl) pyrazinyl-5-oxy)-1,3-dihydro-1-hydroxy-7-methyl-2,1-benzoxaborole (**98**, AN13762) (Figure 8) was chosen as a lead compound, which showed an ED_90_ value of 1.9 mg/kg. The result of the *P. falciparum*-infected mouse model experiment demonstrated that the in vivo parasite clearance profile of **98** was rapid and similar to that of artesunate (water-soluble injectable derivative of ART) and chloroquine, two well-known fast parasite-killing antimalarial medicines [86]. Compound **98** (AN13762) was subjected to potency evaluation against other resistant *P. falciparum* strains, in vivo parasite reduction rate evaluation (or number of parasites the compound could kill in a parasite life cycle, PRR), and for preliminary genotoxicity studies. An in vitro PRR assay against *P. falciparum* was used to compare the parasitic killing rates at different concentrations. The results indicated that the antiparasitic rate of action of **98** was fast and similar to those for ART and chloroquine. Further, **98** was also examined against an additional eleven *P. falciparum* resistant strains which demonstrated high activity with the IC_50_ value in the range of 0.036−0.080 µM, indicating no cross-resistance (Figure 8). Safety studies demonstrated that it was not mutagenic and clastogenic in both the in vitro and in vivo models [86]. Therefore, **98** was further investigated for the development of preclinical studies in humans beginning in 2019 (*MMV-Supported Projects. https://www.mmv.org/research-development*, accessed on 18 January 2021).

**Figure 9:**
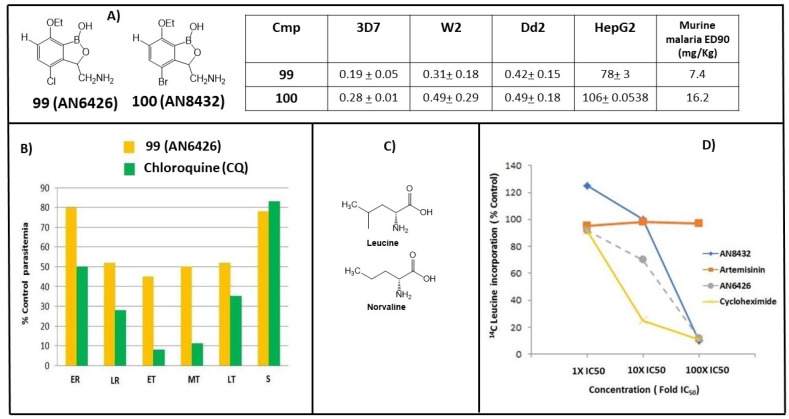
(**A**) In vitro (3D7 and W2/D2d *P. falciparum strains*) and in vivo (*P. bergei strains*) antimalarial activity (IC_50_, µM) of **99** (AN6426) and **100** (AN8432); (**B**) Stage specificity of **99** and CQ; (**C**) Chemical structures of Leucine and analogue Norvaline; (**D**) Effects of benzoxaboroles **99** and **100** and controls ART and Cycloheximide on [^14^C] leucine incorporation by wild-type Dd2 strain *P. falciparum*. (Adapted from [88]).

Compound **98** showed no cross-resistance property and that indicated a possible novel action mechanism or drug resistance of benzoxaboroles that is different from those of CQ and pyrimethamine. The highly electrophilic nature of the boron component of these compounds could lead to interactions with a variety of protein targets via reversible covalent bonds (Figure 1B). The benzoxaboroles **2**, **10** (AN3018), AN3365 and AN3664/ZCL039 (Figure 2C) inhibited bacterial LeuRS in an action mechanism [87,88]. In the course of searching for new antimalarial drugs, a benzoxaborole library of LeuRS inhibitors was screened for potency against cultured multidrug-resistant W2 *P. falciparum* strains and the antimalarial activity was investigated [89]. The two most active 3-aminomethylbenzoxaboroles, **99** (AN6426) and **100** (AN8432) (Figure 9A), were selected and extensively examined. The compounds demonstrated the murine malaria ED_90_ values of 7.4 mg/kg and 16.2 mg/kg for **99** and **100**, respectively, in vivo to *P. berghei*-infected mice. Subsequently, **99** and CQ were investigated in different stages of parasites, and inhibition of parasite development was observed across the life cycle of plasmodium, particularly against trophozoites (Figure 9B) [89]. This inhibition happened only with exogenous norvaline (unnatural amino acid analogue of leucine that is charged to tRNA by LeuRS enzymes), rather than with AN6426-resistant parasites. The results are consistent with a loss of LeuRS editing (Figure 9C). Biochemical studies showed that **99** and **100** caused a dose-dependent inhibition with the incorporation of [14C] leucine, indicative of a block in wild-type protein synthesis (using artemisinin as a negative control and, as a positive control, cycloheximide, protein synthesis inhibitor) (Figure 9D) [88,89]. 

During the screening process, 3-(1-hydroxy-1,3-dihydro-2,1-benzoxaborol-7-yl)-propanoic acid was identified as a potent antimalarial agent against *P. falciparum* asexual blood stage parasites known to be resistant to standard antimalarial drugs [90]. The compound was highly effective when administered orally to treat *P. berghei* (ED_90_: 0.34 mg/kg) and *P. falciparum* (ED_90_: 0.57 mg/kg) infections in mice, with minimal cytotoxicity to mammalian cell lines. Its inhibitory effects were greatest in early to middle trophozoite-stage parasites [90]. Enzyme CPSF-73 is a metallo-*b*-lactamase containing two zinc ions essential in the active site [91]. The *Pf*CPSF3 is a *Plasmodium* homologue of mammalian CPSF-73. Docking calculation of the compound on the *Pf*CPSF3 active site revealed that its terminal carboxylate group, occupying an adjacent phosphate-binding site opposite to R290 and Y252, forms a salt bridge and a hydrogen bond, respectively [90]. The negatively charged tetrahedral oxaborole group was placed at the phosphate position at the cleavage site and it interacted with the two catalytic zinc ions. In these models, the identified *Pf*CPSF3 resistance mutations (T406I, Y408S, T409A and D470N) were found on the *Pf*CPSF3 active site of amino acids interacting with AN3661 [90].

## 4. Neglected Tropical Diseases (NTD)

### 4.1. Trypanosomiasis

Human African trypanosomiasis (also known as African sleeping sickness or HAT), a Neglected Tropical Disease (NTD) that occurs in sub-Saharan Africa, is transmitted to humans through the bite of different species of tsetse fly (*Glossina* spp.). It presents a major threat to the health of more than 57 million people in 36 countries in sub-Saharan Africa [92]. During a blood meal on the mammalian host, an infected tsetse fly injects “trypomastigotes” (a parasitic flagellate protozoa) into skin tissue [93]. The parasites enter the lymphatic system, pass into the bloodstream (stage I, hemolymphatic system) and then transform into bloodstream trypomastigotes, which are carried to other sites (stage II, CNS, central nervous system, spinal fluid). The disease is caused by unicellular *Trypanosoma brucei gambiense (T. b. gambiense),* which is endemic in western and central Africa, or *Trypanosoma brucei rhodesiense (T. b. rhodesiense)*, which is found in eastern and southern Africa [93]. The currently available drugs for the treatments for early-stage infection (stage I) are pentamidine and suramin, while melarsoprol and eflornithine are for late-stage infection (stage II or CNS). All these drugs share the same problems of high cost and toxicity with low efficacy in the late stage and potential development of resistance, and they are not orally bioavailable. Thus, there is an urgent need to develop bioavailable oral treatment with improved efficacy and low toxicity at an affordable cost for the treatment of HAT [92,93]. 

**Figure 10:**
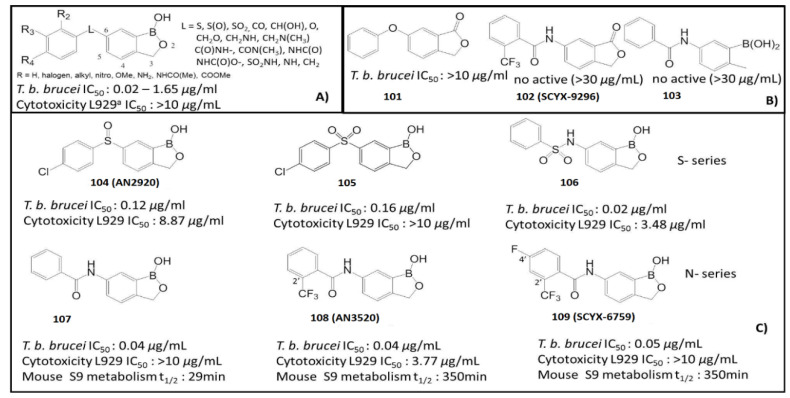
(**A**) Principal linker L in position C(6) of benzoxaboroles; (**B**) Structures and antitrypanosomal activity of no boron analogues **101**–**103**; (**C**) Structures, antitrypanosomal activity, cytotoxicity and biological half-life t_1/2_ of benzoxaborole derivatives S-series **104**–**106** and N-series **107**–**109** (Adapted from [94]).

In 2010, the UCSF Sandler Centre of Drug Discovery, in collaboration with Anacor Pharmaceuticals, identified several compounds through an antitrypanosomal screening of 400 compounds, leading to the discovery of drugs with high potency to inhibit *T. b. brucei*, as shown in Figure 10. Preliminary results of the structure–activity relationships (SAR) suggested that benzoxaboroles containing a substituent at C (6) of the heterocyclic ring system were particularly essential (Figure 10A) [94]. Thus, the oxaborole functionality was crucial for the observed antitrypanosomal activity, as demonstrated by low activity (IC_50_ > 10 μg/mL) or loss of activity upon removal of the oxaborole ring or substitution with carbon (**101–109**) (Figure 10). The length between the hydrogen bond acceptor O and the benzoxaborole C(6) of the linkage group “L” had a significant effect on the antitrypanosomal activity (i.e., in sulfonamide, O-C(6) distance 3.52 Å, IC_50_ 0.02 µg/mL vs. sulfoxide, O-C(6) distance 2.38 Å, IC_50_ 0.17 µg/mL). Compounds with amide linkers showed high potency. Accordingly, the most potent compounds among the series were benzoxaboroles with a sulfonamide linker (**106**) and amide linker (**107**) that showed an improvement in antitrypanosomal activity with an IC_50_ of 0.02 and 0.04 µg/mL, respectively, to inhibit *T. b. brucei* (Figure 10C) [94]. The in vivo assessments using the murine model of blood stage (I) *T. b. brucei* infection showed that the sulfone linker in **105** was more efficacious, with complete cure observed at 20 mg/kg. The sulfonamide linker in **106** exhibited modest in vivo activity with a serious cytotoxicity of 3.48 µg/µL) [95]. By the modification of an amide linked compound, new leads, N-(1-hydroxy-1,3dihydrobenzo[c] [1,2] oxaborol-6-yl)-2-trifluoromethylbenzamide (**108**, AN3520) and 4-fluoro-N-(1-hydroxy-1,3-dihydrobenzo[c] [1,2] oxaborol-6-yl)-2-trifluoromethylbenzamide (**109**, SCYX-6759), were identified (Figure 10C) [95]. These two compounds exhibited high permeability, in vitro metabolic stability (Mouse S9 metabolism t_1/2_ > 350 min), and rapid time-dependent trypanocidal activity against *T. b. brucei*. Pharmacokinetic analysis demonstrated that **108** and **109** were orally bioavailable in multiple species and were able to cross the blood–brain barrier (BBB) at sufficient levels to cure stage II of the HAT disease in mice, with no evidence of interaction with the P-glycoprotein transporter [96]. These oxaborole carboxamides cured stage I (hemolymphatic) trypanosomiasis infection in mice when administered orally at 2.5 to 10 mg/kg of body weight for 4 consecutive days. Metabolism and pharmacokinetic studies in several species, including nonhuman primates, demonstrated that both **108** and **109** were low-clearance compounds [94,95,96].

**Figure 11:**
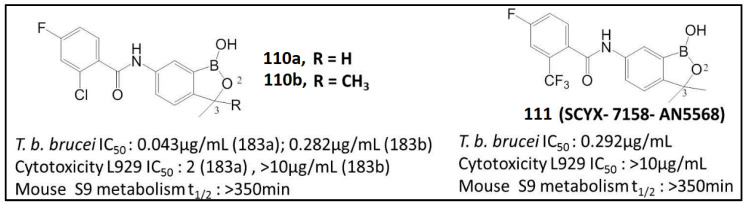
Structures, antitrypanosomal activity, cytotoxicity and biological half-life t_1/2_ of benzoxaboroles **110** and **111** (Adapted from [98]).

Sulfonamide **106** was further modified using various linkers between the heterocyclic core and pendant aryl group to show reasonable potency in the whole-cell *T. b. brucei* assay with low cytotoxicity (IC_50_ > 10 µg/mL for mouse lung fibroblast cells (L929)) [97]. The introduction of a methyl group (**110a**) at C(3) of the benzoxaborole ring had little effect on the trypanocidal potency but caused a significant increase in cytotoxicity (**110a** vs. **110b**), while C(3)-dimethyl analogs (**110b** and **111**) retained trypanocidal activity but were not cytotoxic (Figure 11) [97]. Compound SCYX-7158 (**111**) exhibited enhanced activity against representative strains of *T. b. brucei*, including T. b. *rhodesiense* and *T. b. gambiense* strains (from 0.07 μg/mL to 0.37 μg/mL), following the incubation of the parasite strains with the compound for 72 h [98]. The in vivo activity of these oxaboroles was assessed using the mouse model of acute and chronic HAT. The SCYX-7158 exhibited good permeability across the blood–brain barrier and achieved in measurable levels after both intravenous and oral doses. Phase I assessed the safety, tolerability, pharmacokinetics and pharmacodynamics of SCYX-7158 by applying a single oral ascending dose in 128 healthy human volunteers of sub-Saharan origin. It allowed the therapeutic dose administered at 960 mg once as three tablets, with a favorable safety profile. As the drug has a long half-life (>300 min), the study was extended to 210 days to ensure safety monitoring of the healthy volunteers [99]. Based on the results of this study, DNDi (Drugs for Neglected Diseases Initiative) and partners proceeded to Phase II/III—efficacy and safety study of SCYX-7158 as a single dose oral treatment of patients with HAT [100]. 

**Figure 12:**
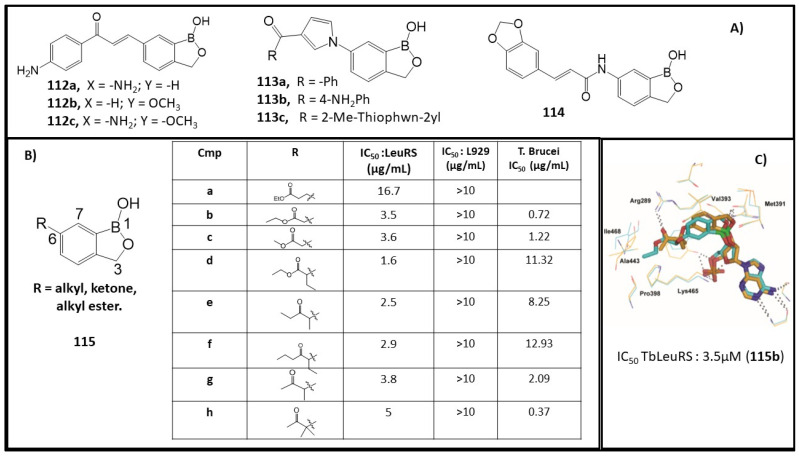
(**A**) Structures of chalcone–benzoxaborole **112**, pyrrolobenzoxaborole **113** and cinnamoyl–oxaborole **114**; (**B**) Structures, antitrypanosomal activity and cytotoxicity of benzoxaboroles **115a**–**h**; (**C**) Binding of ester **115b** (ligand in cyan sticks and binding site residues in cyan lines) in the editing pocket of *Tbb*LeuRS (Adapted from [87,104,105]).

Chalcones have attracted considerable scientific attention and continue to be a versatile scaffold in anticancer and antiprotozoal research. Previously, chalcone-type compounds were found to inhibit the growth of *T. b. brucei* and *Trypanosoma cruzi* parasites [101]. A novel class of chalcone–benzoxaborole hybrid molecules was synthesized and evaluated as an antitrypanosomal agent. The 4-NH_2_ derivative **112a** and 3-OMe derivative **112b** (Figure 12A) were found to have excellent potency against *T. b. brucei* (**112a**, IC_50_: 0.024 µg/μM; **112b**, IC_50_: 0.022 µg/μM) and good cytotoxicity (L929 cells, IC_50_ > 10 μg/mL). The synergistic 4-NH_2_-3-OMe compound **112c** presented a high toxicity (L929 cells, IC_50_: 1.45 μg/mL) [102]. The 6-pyrrolobenzoxaboroles, **113**, represent a new class of potent antitrypanosomal agents. These compounds showed an antiparasitic activity ranging from 0.03 µg/mL to 4.02 µg/mL [103]. Three of the leading compounds (**113a–c**) demonstrated high in vitro activity against *T. b. brucei* (IC_50_: 0.09 µg/mL for **113a**; 0.03µg/mL for **113b**; 0.07 µg/mL for **113c**) and good cytotoxicity (L929 cells, IC50 > 10 μg/mL for **113a** and **113c**). They also showed good possibility to cure the parasitic infection in a murine acute infection model with complete clearance of the parasites in the blood (Figure 12B) [103]. Meanwhile, a set of cinnamoyl–oxaborole amides were also synthesized and screened against nagana *T. b. brucei* for antitrypanosomal activity. Compound **114** emerged as a new hit with an in vitro IC_50_ value of 0.086µM against *T. b. brucei* without inhibitory cytotoxicity against HeLa cell lines (Figure 12A) [104]. 

As discussed before, compound **2** (Figure 1), which is under clinical investigation, was indicated as an antifungal agent by inactivating fungal LeuRS [32]. Encouraged by the inhibitory activity of such compounds, C(6)-ester group-functionalized **115a** and **115b** were synthesized, while **115b** showed a 4-fold improvement in activity (*Tbb*LeuRS IC_50_: 3.5 μM) compared to **188a** (*Tbb*LeuRS IC_50_: 16.7 μM) (Figure 12B) [105]. Compounds **115c–i** were also screened as an effort to improve the stability of the leading ester compounds in vivo while retaining their activity. The addition of methyl or ethyl substituents in the α-position to ketone resulted in a significant enhancement of activity, as demonstrated by compounds **115f–I** (*Tbb*LeuRS IC_50_ 2.5, 2.9 and 3.8 μM, respectively) (Figure 12B). The docking model of compound **115b** showed the formation of a hydrogen bond between its carbonyl and Arg289. The pocket is rather small and hydrophobic, lined by nonpolar amino acid residues including Pro398, Ala443, Ile468, and Ala464, and is a good fit with the terminal ethyl group of the compound **115b**. The docking model of compound **115b** also revealed the existence of space near the carbon of the ester (Figure 12C) [105]. These *Tbb*LeuRS inhibitors showed good potency against the bloodstream form of *T. b. brucei* parasites (*T. b. brucei* IC_50_: 0.37–12.93 μM). Although these substituted ketones exhibited similar enzyme inhibitory activity, the dimethyl ketone derivative, **115h**, showed higher potency (*T. b. brucei* IC_50_: 0.37 μM) than its methyl analogue [105].

### 4.2. Leishmaniasis 

Leishmaniasis is a vector-borne parasitic disease caused by at least twenty species of the genus *Leishmania*, with three main clinical forms of *visceral leishmaniasis* (VL), *cutaneous leishmaniasis* (CL) and mucocutaneous leishmaniasis [106]. This disease is responsible for 700,000 to 1 million new infection cases annually. When an infected female sand fly bites the skin of a person or animal, the Leishmania parasites *promastigotes* (protozoan parasites) are injected into a new host. Once on the skin, *promastigotes* are ingested by phagocytic cells and the parasites differentiate into obligate intracellular *amastigotes*. These parasites replicate and invade other sites of the body. The cycle continues until a sand fly bites the infected individual, taking up some of the *amastigotes* during the process [107]. The absence of effective vaccines gives way to treatment by chemotherapy using drugs such as pentavalent antimonials and amphotericin B as primary control of the disease [108]. However, these drugs require parenteral administration. They are nephrotoxic and an increasing drug resistance in *visceral leishmaniasis* has been identified [109]. The efficacy of the first-line oral drug, miltefosine, has declined rapidly over the past decades due to treatment failure, which results in relapses of the disease [110]. The WHO lists leishmaniasis as one of the NTDs and advocates an urgent need for new, efficient, safe, and affordable drugs for the treatment [111]. 

**Figure 13:**
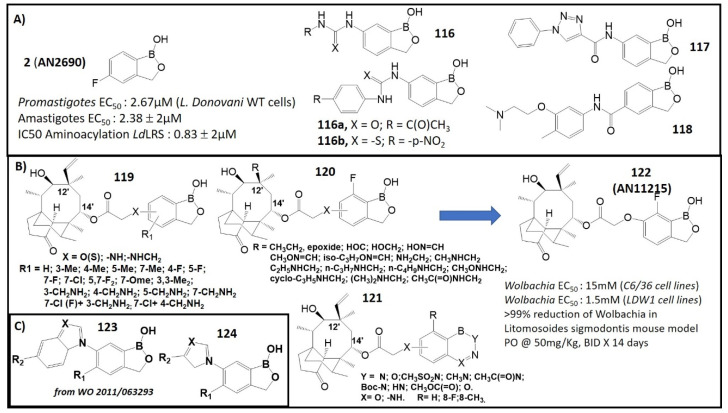
(**A**) Structures and antileishmanial activity of benzoxaboroles **2** and **116–118**(Adapted from [112,115,116]; (**B**) Structures of pleuromutilin–benzoxaboroles **119**–**121** and structure and anti-Onchocerca activity of **122** (Adapted from [117,118]); (**C**) Structures of benzoxaboroles **123** and **124**.

In a new drug screening process, leucyl-tRNA synthetase from *L. donovani* (*Ld*LRS) was selected as a potential drug target for *Leishmania*. This enzyme plays an essential role in the viability of this pathogenic organism and appears to be indispensable for its survival in vitro [112]. Compound **2** (Figure 1) exhibited anti-leishmanial activity against both *promastigote* and *amastigote* stages, in vitro, as well as in vivo in BALB/c mice, as shown in Figure 13A. Moreover, **2** was effective in inhibiting the aminoacylation activity of the recombinant *Ld*LRS (IC_50_: 0.83 ± 0.2 μM), with low toxicity to mammalian cells [112]. Recently, protozoan carbonic anhydrases (CAs) were explored as new targets for drug development for bacteria, fungi and protozoa [113,114]. A type of 6-substituted urea/thiourea benzoxaboroles was tested against CAs from the two pathogenic protozoans (*L. donovani* and *T. cruzi)* [115]. Acetazolamide, a clinically used sulfonamide inhibitor, and Tavaborole **2**, a commercial benzoxaborole used as topical antifungal medication, were used as standard control in the biological assay. The ureido and thioureido benzoxaboroles (**116**) exhibited low micromolar inhibitory activities against protozoans, and their derivative, **116a**, showed the most activity with an inhibition constant Ki of 0.48 μΜ. Compound **116b** containing para-nitrophenyl thiourea exhibited an inhibitory selectivity of 110 times higher towards Leishmania CAs [115]. Compounds **117** and **118**, which showed anti-parasitic activity against *P. falciparum*, *T. brucei*, *T. cruzi* or *L. donovani*, were tested with five different species of *Leishmania* and found to be new leading compounds for its treatment. The efficacy of these drugs, **117** and **118**, was evaluated in vivo against *Leishmania major.* It was found that **117** suppressed lesion growth upon topical application and **118** reduced the lesion size following an oral administration [116].

### 4.3. Onchocerciasis (River Blindness) and Lymphatic Filariasis (Elephantiasis)

Onchocerciasis, also known as “river blindness”, is a parasitic disease caused by the filarial worm *Onchocerca volvulus* and it is transmitted to humans through exposure to repeated bites of infected blackflies of the genus Simulium. Symptoms include severe itching, disfiguring skin conditions, and visual impairment such as permanent blindness. More than 99% of infected people live in African countries [117]. Lymphatic filariasis (commonly known as elephantiasis) is caused by infection with parasite nematodes (roundworms) *Wolbachia. bancrofti* (which is responsible for 90% of the cases), *Brugia. malayi* *and Brugia. timori.* Lymphatic filariasis impairs the lymphatic system and can lead to the abnormal enlargement of body parts, causing pain, severe disability and social stigma. Almost 120 million people in 72 countries worldwide remain threatened by lymphatic filariasis, and they require preventive chemotherapy to stop the spread of this parasitic infection [118]. 

Pleuromutilin and its derivatives are antibacterial drugs through binding to the peptidyl transfer center (PTC) of the ribosomes and consequently inhibiting protein synthesis of the bacteria [119,120]. Jacobs et al. prepared benzoxaborole analogs of the antibiotic type, known as *boronpleuromutilins*, by modification of the pleuromutilin core [121]. This modification was focused on linkers of oxygen, nitrogen and sulfur at the 6-position (Figure 13B). A series of benzoxaborole-incorporated pleuromutilins, **119**–**122**, were tested in in vitro assays in the strain of *Wolbachia*, resulting in encouraging antibacterial potency [121]. Some selected active analogs were analyzed in in vitro absorption, distribution, metabolism, excretion (ADME) and in vivo PK experiments. Compound 7-fluoro-6-oxybenzoxaborole, **122** (AN11251), was identified as a leading compound that showed good in vitro anti-*Wolbachia* activity and physicochemical and pharmacokinetic properties with high exposure in plasma. This compound was effective in reducing the *Wolbachia* parasites following oral administration in mice (Figure 13B). The efficacy of **122** in these models suggests more extensive evaluation of this compound, both alone and in combination with other known anti-*Wolbachia* drugs. Compound **122** may be useful in the treatment of filarial infections or river blindness [121]. In addition, a set of oxaboroles with general structures of **123** and **124** (Figure 13C) were screened against adult worms of *B. malayi* and obtained moderate results [122].

## 5. Cryptosporidiosis and Toxoplasmosis

Cryptosporidiosis, also informally called crypto, is a parasitic disease caused by *Cryptosporidium* *parvum (C. parvum)* species, a genus of protozoan parasites in the phylum Apicomplexa [123]. Cryptosporidiosis causes high morbidity in developing countries [124]. Toxoplasmosis is a disease caused by infection of the *Toxoplasma* *gondii* (*T. gondii*) parasite [125]. The parasite has two distinct life cycles, where the sexual cycle occurs only in cats, and the definitive host and the asexual cycle occur in other mammals and humans. In the human host, the parasites form tissue cysts, most common in skeletal muscle, myocardium, brain, and eyes; these cysts may remain throughout the life of the host [125]. Despite the seriousness of cryptosporidiosis and toxoplasmosis, interest in the development of new drugs targeting these pathogens has been limited. As described previously, aminoacyl-tRNA synthetases (aaRS) play essential roles in protein synthesis and thus they are the suitable targets for antimicrobial drug design for parasitic diseases [126]. Many benzoxaborole compounds designed by this strategy were screened against *Cryptosporidium* to discover new potential drugs. 

**Figure 14:**
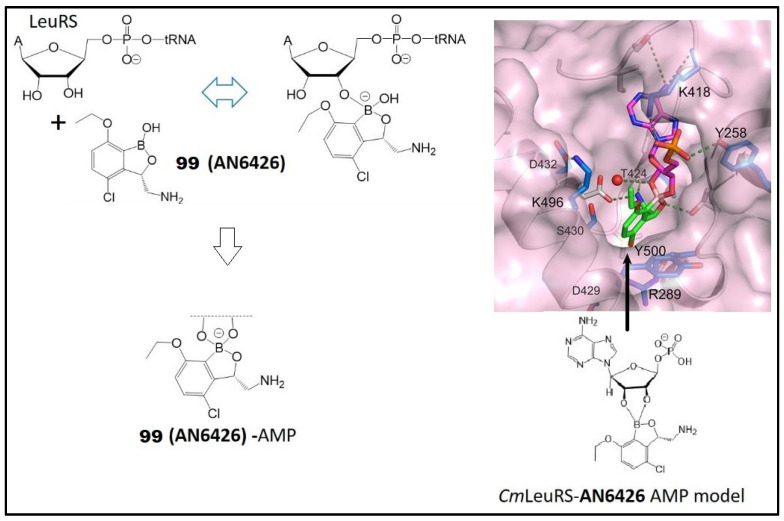
Formation of AN6426-adenosine adduct (**left**) and crystal structures of post-transfer editing analogues and AN6426 with *Cm*LeuRS (**right**). (Adapted from [127]).

Compounds 3-aminomethyl benzoxaborole (**99**, AN6426) and its 4-bromo analogue **100** (AN8432) were found to be active against *C. parvum*, with an IC_50_ value of 2.2 µΜ for **99** and 6.8 μM for **100**, respectively. These activities are comparable to that of nitazoxanide, which is the current standard of care for the treatment of cryptosporidiosis [127]. It was claimed that **99** (AN6426)-AMP adduct can bind to the editing site with a higher affinity than the post-transfer editing substrates (Figure 14). The result was confirmed by in vitro binding experiments and crystal structures of **99** with *Cryptosporidium* leucyl tRNA synthetase (*Cm*LeuRS) [127]. A stable covalent adduct (spiro product) of **99** (AN6426) in the LeuRS editing was formed, and it may block the aminoacylation reaction. These observations were consistent with those of **99** (AN6426) inhibiting protein synthesis in both Cryptosporidium and Toxoplasma by forming a covalent adduct with tRNALeu [127]. Therefore, benzoxaboroles targeting apicomplexan parasites warrant further development in this area.

## 6. Conclusions

Organoboron compounds have been proven to be attractive candidates as pharmaceutical agents because of their unique physical and chemical properties. Besides being used as boron agents in the treatment of boron neutron capture therapy, organoboron compounds are also essential to treat tropical diseases, including tuberculosis and antifungal activity, malaria, neglected tropical diseases and cryptosporidiosis and toxoplasmosis. The current treatments used for tropical diseases are sub-optimal, and in some cases, there are no drugs available to date. Drug resistance for the clinically used antibiotics and anti-protozoan agents is one of the world’s most serious public health problems. In the last few decades, development in the use of boron derivatives as pharmaceutical agents has produced encouraging strides. The clinical introduction of bortezomib as an anti-cancer agent was followed by benzoxaborole drugs, such as tavaborole and crisaborole, for the treatment of onychomycosis and atopic dermatitis. Anti-infective drugs bearing boron atoms in heterocyclic rings represent a highly interesting field for the pharmaceutical industry, with the potential to obtain drugs with a novel mechanism of action which are effective for the management of infective diseases. It is essential to engage research on anti-tropical diseases with a distinct schedule of short, medium and long-term strategies. This is particularly challenging at the present time, since the COVID-19 crisis has significantly shifted both research attention and governmental resources, and that limited human and financial resources can be used to address such tropical diseases and other diseases [128,129]. 

## Data Availability

Not applicable.

## Abbreviations

*Mycobacterium tuberculosis*	*Mtb*
H. proteasome	Human proteasome
MIC	Minimum inhibitory concentration
ACT	Artemisinin-based combination therapies
aaRSAmino	Acyl tRNA synthetase
CQ	Chloroquine
ADME	Absorption, distribution, metabolism, and excretion
SsrA-tagged protein	Caseinolytic-protease-specific degradation protein
RLU	Relative luminescence
WT cell lines	Wild-type cell lines
aaRS	Aminoacyl-tRNA synthetase
ClpP	Caseinolytic proteases
TB	Tuberculosis
Chloromethyl ketones	CMKs
enoyl- reductase	ENR
Enoyl-[acyl-carrier-protein] reductase [NADH]	InhA
Nicotinamide adenine dinucleotide oxidized	NAD+
Nicotinamide adenine dinucleotide reduced	NADH
artemisinin-based combination therapies	ACT
*Plasmodium Falciparum*	*P. Falciparum*
*Plasmodium falciparum 3D7*	CQ-sensitive 3D7
structure−activity relationship studies	SARs
3D7	Chloroquine (CQ) sensitive *P. falciparum* strain
W2	Chloroquine (CQ) resistant *P. falciparum* strain
D2d	Chloroquine (CQ) resistant *P. falciparum* strain
half-life	t1/2
availability	F
*WT* cells	Wild-type cells

TOF
